# USING A “TEAMING” (ENHANCED SOCIAL SUPPORT) APPROACH IN THE CONTEXT OF THE RISE ELDER ABUSE AND SELF-NEGLECT INTERVENTION

**DOI:** 10.1093/geroni/igad104.1154

**Published:** 2023-12-21

**Authors:** Andie MacNeil, M T Connolly, Erin Salvo, Patricia Kimball, Geoff Rogers, Stuart Lewis, David Burnes

**Affiliations:** University of Toronto, Toronto, Ontario, Canada; University of Southern California, Los Angeles, California, United States; Department of Health and Human Services, Augusta, Maine, United States; Elder Abuse Institute of Maine, Brunswick, Maine, United States; City University of New York, New York City, New York, United States; Dartmouth College, Lebanon, New Hampshire, United States; University of Toronto, Toronto, Ontario, Canada

## Abstract

Our understanding of what intervention strategies are effective in improving the well-being of older adults experiencing elder abuse and self-neglect (EASN) is severely limited. However, data consistently demonstrate that social support is a protective factor. As a component of a larger community-based EASN intervention, RISE, this study examined the use of a method called “teaming,” a wraparound approach to establish sustained formal and informal social supports surrounding victims and alleged harmers in EASN cases. Qualitative interviews and a focus group were conducted with the original pilot cohort of RISE “advocate” caseworkers (n = 4). A descriptive phenomenological approach involving two independent assessors was used to code transcripts into themes. Three domains were identified: (1) team and support forming process, which describes the development of a supportive network based on each client’s needs; (2) techniques, which refers to the specific strategies advocates utilized to promote collectivity and shared responsibility around the client; and (3) implementation challenges, which discusses the difficulties advocates encountered when using teaming with people experiencing EASN. The experiences of advocates suggest that teaming is a beneficial approach to support the individualized needs of each client, and to promote improved and sustainable case outcomes for clients. This study represents the first in-depth exploration of teaming in the context of EASN intervention.

